# The Role of Polyphenols in Abiotic Stress Response: The Influence of Molecular Structure

**DOI:** 10.3390/plants10010118

**Published:** 2021-01-08

**Authors:** Dunja Šamec, Erna Karalija, Ivana Šola, Valerija Vujčić Bok, Branka Salopek-Sondi

**Affiliations:** 1 Ruđer Bošković Institute, Bijenička cesta 54, 10000 Zagreb, Croatia; salopek@irb.hr; 2 Faculty of Science, University of Sarajevo, Zmaja od Bosne 33–35, 71000 Sarajevo, Bosnia and Herzegovina; erna.karalija@gmail.com; 3 Department of Biology, Faculty of Science, University of Zagreb, Horvatovac 102a, 10000 Zagreb, Croatia; ivana.sola@biol.pmf.hr (I.Š.); valerija.vujcic@biol.pmf.hr (V.V.B.)

**Keywords:** abiotic stress, polyphenols, phenolic acids, flavonoids, stilbenoids, lignans

## Abstract

Abiotic stressors such as extreme temperatures, drought, flood, light, salt, and heavy metals alter biological diversity and crop production worldwide. Therefore, it is important to know the mechanisms by which plants cope with stress conditions. Polyphenols, which are the largest group of plant-specialized metabolites, are generally recognized as molecules involved in stress protection in plants. This diverse group of metabolites contains various structures, from simple forms consisting of one aromatic ring to more complex ones consisting of large number of polymerized molecules. Consequently, all these molecules, depending on their structure, may show different roles in plant growth, development, and stress protection. In the present review, we aimed to summarize data on how different polyphenol structures influence their biological activity and their roles in abiotic stress responses. We focused our review on phenolic acids, flavonoids, stilbenoids, and lignans.

## 1. Introduction

In the last couple of decades, more scientific evidence has been found to support the fact that climate and atmospheric changes can rapidly alter biological diversity [1] and crop production [2] around the world. Environmental stress can be defined as any change in growth condition(s) within the plant’s natural habitat that alters or disrupts its metabolic homeostasis. In general, we recognize two different types of stress: biotic (caused by insects, bacteria, or viruses) and abiotic. Abiotic stressors such as extreme temperatures, drought, flood, light, salt, and heavy metals largely influence plant development and crop productivity.

Plants, as sessile organisms, respond to stress conditions with changes in the gene expression pattern of proteins that control the biosynthesis of metabolites involved in interactions between a plant and its environment. Polyphenols are an important class of specialized metabolites that play crucial physiological roles throughout the plant life cycle, including responses to stress. It is well known that the phenylpropanoid biosynthetic pathway is usually activated under harmful environmental conditions such as drought, extreme temperatures, salinity, heavy metal pollutions, and ultraviolet radiations, resulting in the accumulation of various phenolic compounds [3,4].

**Figure 1:**
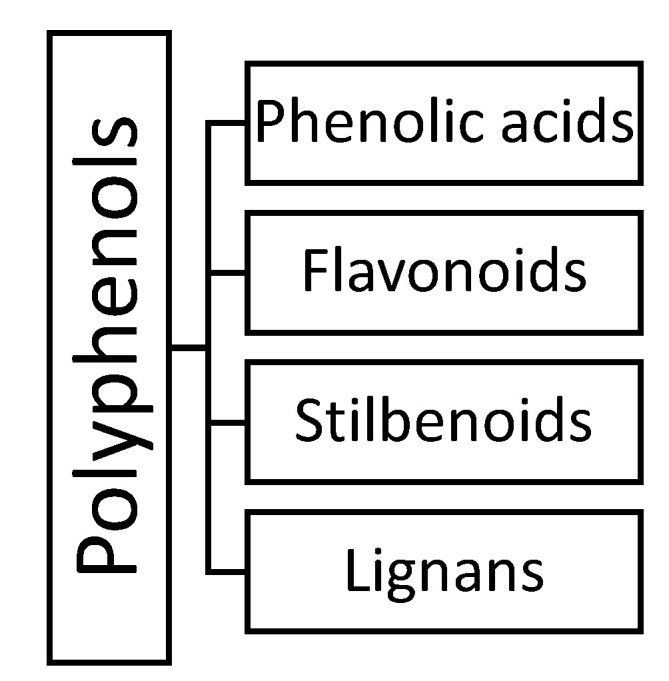
The basic division of the phenolic compounds.

Polyphenols are the largest and the most studied group of plant-specialized metabolites, which include more than 8000 molecules [5]. They are all biosynthesized through a shikimate/phenylpropanoid pathway that produces a wide array of monomeric and polymeric polyphenols [3]. The structure of phenolic compounds varies extensively, although their common feature is the presence of one (simple phenolics) or more (polyphenols) hydroxyl substituents, attached directly to one or more aromatic or benzene rings. According to their structures, they may be grouped into phenolic acids, flavonoids, stilbenoids, and lignans (Figure 1). In general, phenolic compounds may be present in plants as free forms, but more often, they are found in conjugated forms with one or more sugar residues linked by β-glycosidic bonds to a hydroxyl group (O-glycosides) or a carbon atom of the aromatic ring (C-glycosides). The associated sugars can be monosaccharides, disaccharides, or even oligosaccharides [5].

Polyphenolic compounds are often considered as a group of molecules with similar biological activity, and especially in biological studies, researchers measure total polyphenol content as a marker of biological activity. However, the structure of polyphenols significantly influences their activity and role in biological processes, and consequently, their involvement in plant stress responses. Our review aims to provide a comprehensive summary of the currently available literature on how different polyphenolic structures (phenolic acids, flavonoids, stilbenoids, lignans) influence plant stress responses.

## 2. Phenolic Acids

**Figure 2:**
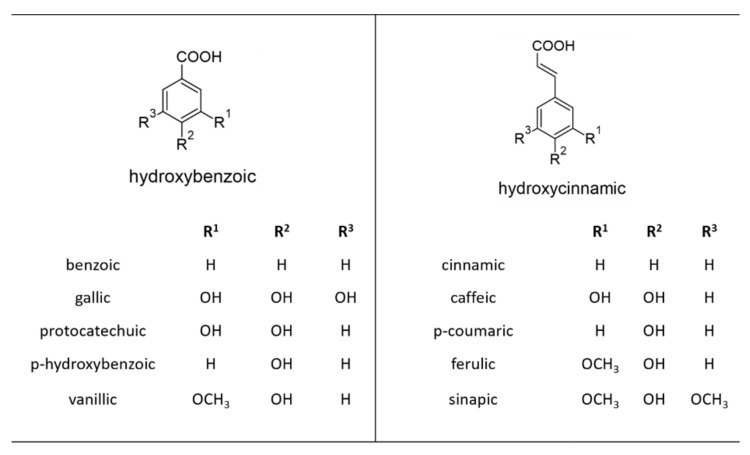
The structures of the major phenolic acids.

A carboxyl group attached or linked to a benzene ring is a main feature of all phenolic acids. They are rarely found in free form, and are often linked by ether, acetal, or ester bonds to structural components of a plant cell (cellulose, proteins, lignin), other smaller organic molecules (e.g., quinic, maleic, or tartaric acids, and glucose), other larger polyphenols (flavonoids), or natural products (e.g., terpenes) [6,7]. Depending on their structure, phenolic acids can be distinguished into two classes: derivatives of benzoic acid (hydroxybenzoic acids, C6–C1) and derivatives of cinnamic acid (hydroxycinnamic acids, C6–C3) (Figure 2) [8].

Hydroxybenzoic acids (HBAs) are derived from benzoic acids such as *p*-hydroxybenzoic acid (p-HBA), producing salicylic acid (SA), gallic acid (GA), vanillic acid (VA) and variations of dihydroxybenzoic acid (2,3-DHBA; 2,5-DHBA; 3,4-DHBA; 3,5-DHBA) [8]. Variations in the structure of HBAs are the result of hydroxylations and methylations of the aromatic ring in their structure [9]. Some of the simple HBAs can be formed from intermediates early in the shikimate pathway, including gallic acid, which is derived from dehydroshikimic acid. The mechanisms and all the enzymes involved in the biosynthesis of HBAs and their derivatives are still not completely known [8].

Hydroxycinnamic acids (HCAs) include *p*-coumaric, caffeic, ferulic, and sinapic acids, and their esterified/etherified conjugates (e.g., chlorogenic acid). The conjugated forms are esters of hydroxyl acids such as quinic, shikimic, and tartaric acid, and their sugar derivatives [10]. The formation of caffeic, ferulic, 5-hydroxyferulic, and sinapic acids (from *p*-coumaric acid) requires hydroxylation and methylation. The addition of a second hydroxyl group into *p*-coumaric acid is catalyzed by monophenol mono-oxygenases [11]. Methylation of caffeic acid leads to the formation of ferulic acid, which, together with *p*-coumaric acid, is a precursor of lignin [8]. Lignin is an important natural polymer that may play crucial roles in abiotic stress in plants. For example, integrated analysis of transcriptomic and metabolomic data reveals that chilling stress increases the number of polyphenols, especially lignin, which protects tobacco from the harmful effects of low temperature [12].

Plant phenolic acids are powerful antioxidants that can mediate scavenging of harmful reactive oxygen species (ROS) in plants under different abiotic stressors [13,14]. The activation of a plant’s antioxidant system is a result of the stimulation of the phenylpropanoid biosynthetic pathway, inducing synthesis of phenolic acids [13,15] such as caffeic, cinnamylmalic, gallic, ferulic, and vanillic acids [13,16,17]. The accumulation of endogenous phenolic acids as a mechanism of plant tolerance against abiotic stress, including temperature, nanoparticles, and pesticides, has been recorded in many plant species [18,19,20,21]. The key genes phenylalanine ammonia lyase (PAL) and chalcone synthase (CHS) are up-regulated under abiotic stress, resulting in increased phenolic biosynthesis [19,20], with a final result of enhanced tolerance of abiotic stress factors [21,22].

The antioxidant capacity of phenolic acids and their derivatives is mainly related to the number of hydroxyl groups. The monohydroxybenzoic acid isomers *p*- and *o*-hydroxybenzoic acids show positive charge on their carboxyl group, while in meta isomers this charge is absent, resulting in much easier oxidation of *m*-hydroxybenzoic acids [23]. The carboxyl group itself does not have electron-scavenging abilities, although deprotonated carboxyl becomes electron-donating and group-favoring in the transfer of an H atom, resulting in electron-donating radical scavenging activity [24]. The antioxidant potential and role of HBA derivatives in stress response in plants has been investigated for different types of stress. An increase in vanillic acid has been observed in *Zea mays* under Cu, Pb, and Cd stress [25], and in *Cucumis sativus* under drought stress [26]. Strong antioxidant properties of vanillic acid have been reported recently [27]. Studies on tomatoes showed that the exogenous application of vanillic acid can enhance salt tolerance by strengthening the osmolyte accumulation (such as proline), ion regulation (increase in K, Ca, and Mg) and antioxidant activities (increase in superoxide dismutase (SOD), catalase (CAT), and ascorbic acid (AsA)) [28]. Similar alleviating effects on salt-induced osmotic stress have been recorded for ellagic acid, a condensed dimeric product of gallic acid, a dilactone containing four hydroxyl groups [29]. The biological activities of ellagic acid have been investigated since the late 1990s [30]. Hydroxyl groups and lactone structures in ellagic acid are rich in hydrogen bonds, and can act as electron acceptors and hydrogen donors with the ability to accept electrons from different substrates, making ellagic acid a powerful scavenger [30]. Furthermore, ellagic acid can work as a primary oxidant (as a free-radical scavenger) and as a secondary antioxidant (e.g., indirect pathways, metal chelation, decomposition of hydro-peroxide, and deactivation of singlet oxygen), making this acid a multiple-function antioxidant [31]. An increase in several HBAs (gallic, vanillic, syringic, *p*-hydroxybenzoic, and ellagic acids) in *Amaranthus tricolor* under salt stress was recorded [32]. Three of the above-mentioned HBAs are constituents of lignin (*p*-hydroxybenzoic, vanillic, and syringic acids). Increased lignification is often a plant’s response to biotic and abiotic stress conditions. Lignin-enriched tissues can serve as a barrier that limits metal uptake or prevents pathogen entry [33,34]. For plant growth, development, and stress protection, the HBA salicylic acid is important, and this acid is recognized as a plant hormone [35]. Changes in salicylic acid level have been reported under different abiotic stress conditions [4,36], and the application of salicylic acid has been shown to be beneficial for plants under either normal or stress conditions [37]. As reviewed by Khan et al. [35] and Hernández-Ruiz [37], salicylic acid can regulate various plant metabolic processes, modulate the production of varied osmolytes and specialized metabolites, and maintain the nutrient status of plants, thereby protecting them under abiotic stress conditions.

HCAs are often more effective antioxidants than HBAs [38]. Polymerization of *p*-coumaric, ferulic, and sinapic acids and their alcohols leads to the formation of lignin. Under stress condition, such as Cu or Cd stress, the accumulation of lignin, premature lignification of roots, and suberin deposition have been recorded [39,40]. It has been suggested that esterification of phenolic acids forms lignin-like polymers that increase the number of lignin attachment sites, indicating that esterification and lignification are continuous processes [33]. Such findings indicate that phenolic esters play a predominant role in lignin synthesis [41] and the stress response of plants to heavy metals. Plant exposure to Cd can increase *p*-coumaric acid in glycoside-bound and cell-wall-bound fractions, while in the case of Cu exposure, an increase in ferulic acid was recorded. It was noted that there is nonspecific esterification in cell walls that could be related to resistance against heavy-metal stressors [33].

The antioxidant potential of HCAs and their derivatives is also related to the availability of hydroxyl groups in the molecules [42]. Antioxidant potential is enhanced with more hydroxyl groups and methoxylation (e.g., accumulating the ferulic acid is more protective than *p*-coumaric acid) [38]. Esterification, especially dimethyl esterification of 8,5-benzofurandiferulic acid, can decrease antioxidant properties in hydroxycinnamates. The effects of dimerization on the antioxidant potential of hydroxicinnamates depend on the nature of the linkage, the number of phenolic hydroxyl groups, the presence of alkyl chains, and the number of sites that can scavenge unpaired electrons [43]. The increased accumulation of chlorogenic acids (CGAs) and related esters (included in lignin biosynthesis) in plants under biotic and abiotic stress has been observed in many species [15,21,32]. CGAs are derived by esterification of caffeic, ferulic, and *p*-coumaric acids with quinic acid, and the resulting conjugated structures can exist in several isomeric forms. CGAs contain vicinal hydroxyl groups on aromatic residues [44]. The antioxidant potential of CGAs is result of their oxidation to respective phenoxyl radicals that are quickly stabilized. Pro-oxidative activity of CGAs can be initiated by the presence of transition metals (Cu, Fe) [44]. Ferulic acid is another HCA that experiences a level change under abiotic stress. Ferulic acid, besides its ability to act as a free-radical scavenger, can also inhibit enzymes involved in free-radical generation, and it can enhance the activity of other scavenging enzymes [45]. Ferulic acid can chelate protonated metals such as Cu(II) and Fe(II) [38] by directly binding to these metals [46] and preventing the formation of hydroxyl radicals and cell-membrane peroxidation [47]. The antioxidant activity of this phenolic acid is related to its structure [48] and its ability to form stable phenoxyl radicals in reaction with radical molecules. It has also been proposed that ferulic acid acts as hydrogen donor that protects cell membranes from the auto-oxidation process. Two soluble HCAs—ester bound chlorogenic acid and glycoside bound, *p*-coumaric acid-O-glucoside—are often accumulated under Cd stress. The accumulation of soluble HCAs has been recorded in *Crotalaria juncea* [49]. The response of the shikimate pathway to different metals differs (e.g., in the case of Cu, an increase in the activity of dehydrogenase and peroxidase was recorded, while in the case of nickel stress, an increase in PAL activity was recorded) [50]. It seems that plant responses to heavy-metal stress are metal-specific, and could be related to the physical properties of the metal itself, resulting in different triggering of metabolic pathways within phenolic acid synthesis [51].

Gallic acid, together with ellagic acid, is a substituent of polyphenolic biomolecules called hydrolyzable tannins (HT) [52]. These compounds, as the name suggests, are hydrolyzed by weak acids, and can be divided into gallotannins, which provide sugar and gallic acid, and ellagitannins, which, in addition, yield ellagic acid after hydrolysis [53]. The main function of these compounds in plants is to provide protection against microbial pathogens, harmful insects, and other herbivores [52], but as summarized by Furlan et al. [54] they are synthesized in plants in response to the influence of environmental stressors such as drought, UV-B radiation, and atmospheric pollution. These effects result from the ability of tannins to bind proteins, to act as antioxidants or pro-oxidants, and to chelate iron and other metals [55]. Tannins generally contain ortho-dihydroxyl substitution patterns, and can chelate metals and help to protect plants against heavy-metal toxicity [55]. They also possess an allelopathic effect. [55].

## 3. Flavonoids

**Figure 3:**
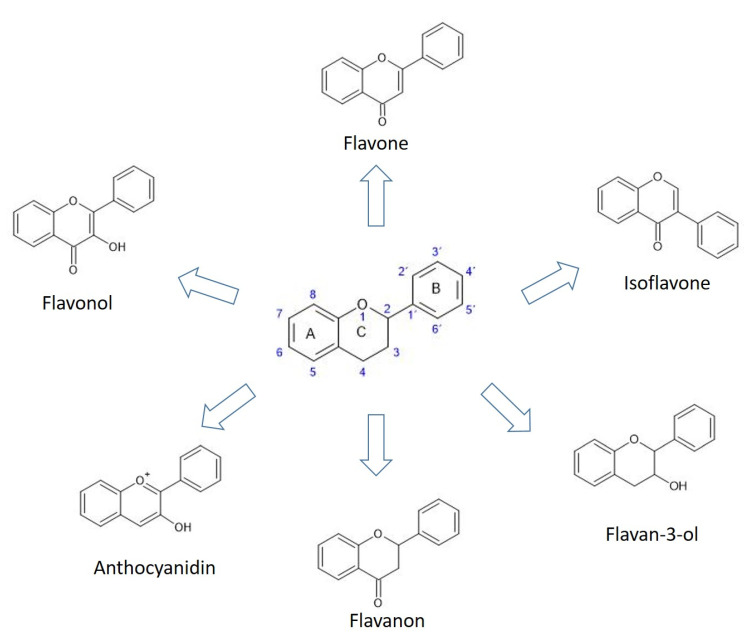
The basic division of the flavonoids.

Flavonoids, which are the most commonly studied group of polyphenols, have more than 6000 different structures [56]. Based on their structure, flavonoids can be divided into six groups: flavones, flavonols, flavan-3-ols, flavanonols, flavanones, isoflavones, and anthocyanins, which differ in the pattern of their central heterocyclic pyrane ring of core flavan structure (Figure 3). The carbon atoms in flavonoid molecules form two benzene rings (A and B), which are connected by three carbon atoms and one oxygen atom, forming a central pyrane ring (C) (Figure 3).

Flavonoids may be present in plants in free form, but are more commonly derivatives that are synthesized by processes such as glycosidation, prenylation, acetylation, methylation, and polymerization, which affects their bioactivity [57]. The most common form of flavonoid derivatives are glycosides, frequently O-glycosides, and less frequently, C-glycosydes [58]. Modification may involve a single oligosaccharide, or in some cases, a polysaccharide moiety. Glycosidation improves solubility, biodistribution, and metabolism by enabling transport across cell membranes [59]. Glycosides serve as a storage forms for metabolites, and may be involved in detoxification processes [59]. Methylated flavonoids are less common than free forms or flavonoid glycosides [57]. C-methylation and O-methylation are two common methylation patterns of flavonoids. Methylation has been documented to enhance the entry of flavonoids into cells and to prevent the cells’ degradation [57].

The most commonly investigated biological process of flavonoids is antioxidant activity. In general, as summarized by Williamson et al. [43], most of the free flavonols are efficient antioxidants in both the aqueous and lipid phases, but increased hydroxyl groups on the B ring tend to increase activity. Glycosylation of flavonoids decreases their antioxidant activity, but the effect is much more marked when the substitution is in the B-ring (as a 4′-substitution). The antioxidant activity tends to decrease with an increasing number of sugar moieties on one position.

### 3.1. Flavanones

**Figure 4:**
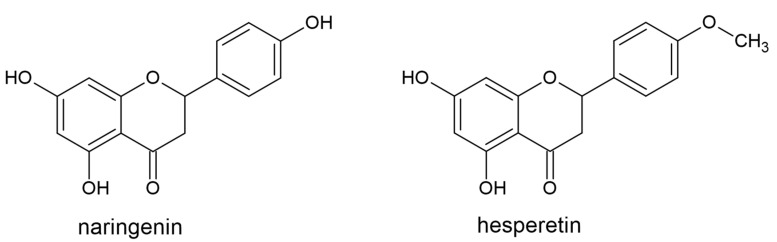
The structures of the flavanones naringenin and hesperetin.

Chalcone isomerase and flavanone-4-reductase use a chalcone-like compound and flavan-4-ol as a substrate, respectively, to produce flavanones. Flavanones, or dihydroxyflavones (hesperetin, naringenin, eridictyol, sylibin, isosakuratenin), have reduced C2 and C3 in the C ring, and therefore do not contain a double bond in the C-ring (Figure 4). They are the precursors of all other flavonoid classes [60]. Flavanones are highly reactive, and they have been reported to undergo hydroxylation, glycosylation, and O-methylation reactions [61]. The most common flavanone derivatives in nature are glycosides [60], which most often contain glucose or disaccharide at C7. They are recognized as important phytochemicals in citrus fruits [62]. However, flavanones are present in smaller concentrations in other plant species as well.

Flavanones are involved in biotic interactions [63], although their role in abiotic stresses have been investigated as well. In tomatoes, heat stress induced the accumulation of naringenin and naringenin chalcone, whereas under salinity or the combination of salinity and heat, the same compounds were downregulated compared to the control [64]. Heat stress and a combination of heat and salinity showed almost the same intensity effect, but in an opposite manner, suggesting that the central pyrane ring in a flavonoid structure is not decisive in a response to heat. Gamma irradiation also reduced the concentration of naringenin in tomatoes [65]. The concentration of hesperidin was significantly increased by UV-C radiation in *Cyclopia subternata* (honeybush) callus [66]. UV-B treatment of peppermint (*Mentha* × *piperita*) plants, grown in both fields and growth chambers, enhanced the concentration of hesperidin. Narirutin (naringenin-7-O-rutinoside), on the other hand, was decreased in fields, but not affected in growth chambers [67]. This might suggest the relevance of the methoxy group in flavanone structures in response to UV stress. Heat drying (at 60 °C and 90 °C) of leaves of *Salix purpurea* reduced the amount of naringenin-7-O-glucoside, and even more significantly, the amount of eriodictyol-7-O-glucoside [68]. This suggests that a flavanone with two hydroxyl groups on its B ring is more susceptible to heat than one with one hydroxy group. For comparison, freeze-drying and room drying showed the same effect on these two compounds. Cold stress increased or decreased naringin level in different rosemary accessions [69].

Soil flooding increased concentrations of eriodictyol glycosides in leaves of citrus genotypes [70]. A water deficit did not affect hesperidin levels, while naringin levels increased significantly in leaves of *Citrus unshiu* [71]. To the contrary, in peppermint, hesperidin was significantly increased, while aglycone naringenin only slightly increased under water deficit [72]. Transgenic tobacco (*Nicotiana tabacum*) plants with an overexpressed CHS gene showed an increased concentration of naringenin, and especially naringin, and were more tolerant than the control plants to drought stress [73]. This suggests a higher relevance of glycosylated naringenin than aglycone under drought stress. Since naringenin exhibits a higher antioxidant capacity and hydroxyl and superoxide radical scavenger efficiency than naringin [74], the response to drought stress is not primarily accomplished via the antioxidant system. Glycosylation attenuated the efficiency in inhibiting the enzyme xanthine oxidase, and the aglycone could act like a more active chelator of metallic ions than the glycoside [74].

High sodium concentration in the nodules reduced the concentration of naringenin in chickpea (*Cicer arietinum* L.) [75]. In combination with arbuscular mycorrhiza, naringenin ameliorated the negative effects of salinity, suggesting the importance of the naringenin/arbuscular mycorrhiza combination in improving the symbiotic efficiency of chickpea under salt stress [75]. The treatment of roots with the flavonoids hesperetin or naringenin stimulated plant–fungus interactions during the precolonization. Exogenous naringenin diminished the effect of salt and osmotic stresses on bean (*Phaseolus vulgaris*) plants’ photosynthetic activity and chloroplast antioxidant system [76,77] as well.

CdCl_2_-treatment increased the concentration of naringenin in turnip (*Brassica rapa* ssp. *rapa*) plants, while hesperidin was not affected [78]. Exogenous naringenin alleviated Pb induced morphological and biochemical alterations in mung bean (*Vigna radiata*) [79]. These results indicate the relevance of naringenin in plant responses to toxic metals.

Naringin and hesperidin increased in peppermint treated with salicylic acid compared to controls [80]. However, H_2_O_2_ did not affect the hesperidin level.

### 3.2. Flavones

**Figure 5:**
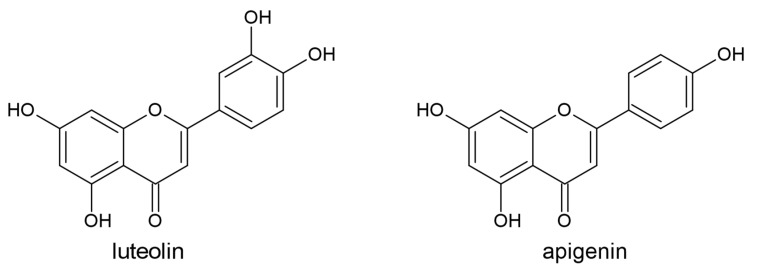
The structures of the flavones luteolin and apigenin.

Flavones (luteolin, apigenin, chrysin, baicalein, tangeritin, diosmetin, orientin, and scoparin) contain a double bond between the atoms C2 and C3, and a ketone group on the atom C4. They are synthesized from flavanones by means of flavone synthase (FNS), which catalyzes the oxidation of C2 and C3 atoms and the formation of the double bond between them (Figure 5).

The most emphasized characteristic of flavones in plants is their antioxidant activity. For example, it is well established that a flavone with two ortho-hydroxy groups on the B ring (luteolin) is a better electron donor than a flavone with one hydroxy group (apigenin). This means that dihydroxy B-ring-flavones scavenge stress-induced free radicals more effectively than those with one hydroxy group. The accumulations of luteolin and apigenin were reported to be inverted in several experiments. For example, under drought, luteolin levels in the leaves of Chrysanthemum cultivars increased, while the level of apigenin decreased or did not change [81]. Similar, a combination of flooding and excess salinity caused an increase in the luteolin level in leaves of artichoke and cardoon, but did not affect the apigenin level [82]. In chamomile (*Matricaria recutita*), in which apigenin is a bioactive compound important to the pharmaceutical industry, irrigation with saline had no significant effect on the apigenin content [83]. In black cumin (*Nigella sativa*) seedlings, salinity stimulated the biosynthesis of apigenin [84]. An exogenous application of apigenin alleviated the negative effects of salinity on rice seedlings [85]. These examples show that the role of flavones in stress response probably depends on the plant species, or even the cultivar. The level of flavones in plants also may depend on the time of stress exposure. For example, in *Achillea pachycephala*, during the first 14 days, luteolin and apigenin exerted different patterns of concentration change; however, upon 21 and 28 days of drought, their concentrations increased in a similar way [86]. This could be because, at the beginning of drought stress, plants intensively increase their concentration of more effective defense molecules (luteolin compared to apigenin) to adjust to the environment, while later they induce both flavone types equally. However, this may not be the case in all plants. For example, in *Dracocephalum kotschyi* under salt stress, the authors did not observe different patterns in apigenin and luteolin accumulation [87].

In addition to presence in free form, flavones are present in plants as glycosides. In *Achillea pachycephala* under the drought, apigenin-7-*O*-glucoside content decreased, while luteolin-7-*O*-glucoside increased [86]. High light irradiance (with UV-A and UV-B) induced the biosynthesis of dihydroxy B-ring-substituted flavonoids (luteolin-7-O- and quercetin-3-*O*-glycosides), but did not affect the biosynthesis of monohydroxy B-ring-substituted flavonoids (apigenin-7-*O*- and kaempferol-3-*O*-glycosides) [88,89]. This could be due to the fact that quercetin and dihydroxyflavones have greater abilities to scatter harmful UV-B radiation than monohydroxyflavones [90].

In *Psilotum nudum* L., a plant that was traditionally thought to be descended from the earliest vascular plants, a large number of less common *C*-glycosides and dimers of the flavone apigenin were recently identified [91]. This could indicate that *C*-glycosilation and dimerization are processes involved in abiotic stress response, because these compounds may have helped *P. nudum* survive thousands of years and several epochs of changing climate. However, this must be confirmed in the future.

### 3.3. Isoflavones

**Figure 6:**
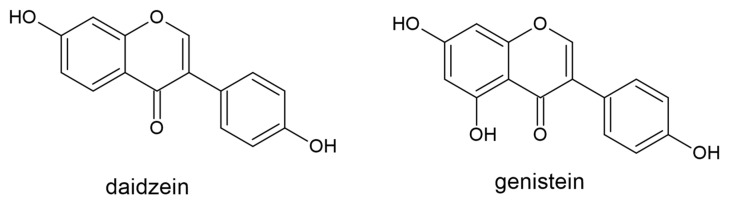
The structures of the isoflavones daidzein and genistein.

Isoflavones are a group of isoflavonoids primarily found in legumes [92]. First, liquiritigenin and naringenin are hydroxylated by 2-hydroxyisoflavanone synthase (IFS) and 2-hydroxyisoflavone dehydrate (HID) to form daidzein, genistein (Figure 6), and formononetin. These isoflavone scaffolds are then further modified by glycosyltransferases and methyltransferases, yielding diverse isoflavonoids [93]. Isoflavones have garnered much scientific attention due to their estrogen-like properties, but a critical evaluation of clinical studies has created some controversy regarding their efficacy [94].

In plants, isoflavones are involved in various plant–pathogen interactions [95], but they are also reported to be involved in abiotic stress responses. Their levels under stress depend on the studied plant and stress type. Several studies have reported that UV-A and UV-B light have a positive influence on isoflavone accumulation [96,97,98], although some other stress reports are not unambiguous. For example, in a study by Swigonska et al. [99], after long and short cold stress, osmotic stress, and combined cold and osmotic stress, the content of all identified isoflavones (daidzin, genistin, deidzein, genistein) increased in roots of soybean seedlings. However, some studies showed that drought causes a decrease in the isoflavones content of soybeans [100]. Gutierrez-Gonzalez et al. [100] found that long-term progressive drought significantly decreased the total isoflavone content in soybean seeds during most of the seeds’ developmental stages, and that the enzyme isoflavone synthase 2 was a major contributor to the reduction of isoflavones under drought. A reduction in isoflavone content in soybeans at late reproductive stages under high temperature stress was also reported [101]. Contrary to this, low temperature was reported to increase the accumulation of two major isoflavones, calycosin and its 7-*O*-β-D-glucoside, in different tissues of *Astragalus membranaceus* var. *mongholicus* seedlings [102]. Isoflavone accumulation was reported to be a marker of salt tolerance, showing a decrease in the salt-tolerant soybean cultivar and an increase in most parts of salt-sensitive cultivar under salt-stress conditions [103]. Furthermore, ozone treatment [92,104] and heavy-metal stress induced isoflavone content [105].

Glycosylation and malonylation are important modifications of isoflavonoids that may stabilize them, enhance their solubility, and facilitate their transport or storage [95]. Arora et al. [106] compared the in vitro antioxidant power of the naturally occurring glycosilated and methoxylated forms of isoflavones with that of their free aglycones. They reported that isoflavonoids were more potent inhibitors of peroxidation caused by metal ions compared to peroxidation caused by peroxyl radicals. Regarding the structure, the number and position of hydroxyl groups was found to be an important determinant of antioxidant activity. Hydroxyl groups were found to be of critical importance at the C-4 position, of moderate importance at the C-5 position, and of negligible importance at the C-7 position. The loss of the 2,3-double bond coupled with the absence of the 4-oxo group conferred the greatest antioxidant activities to these compounds.

Glycosilation of isoflavones is reported to be a common modification under stress conditions, but its mechanism is not well explained. It was reported that UV-B stress could induce glycosylation process in two Astragalus plants [97]. Malonylation is other important process of isoflavones under stress. Malonylated isoflavones are the major forms of isoflavonoids in soybean plants [95]. Ahmed at al. [95] showed that the isoflavone malonyltransferases known as glycine max isoflavone malonyltransferase 1 (GmIMaT1) and glycine max isoflavone malonyltransferase 3 (GmIMaT3) differently modify isoflavone glucosides in soybeans (*Glycine max*) under cold (4 °C), heat (42 °C), drought stress, low pH (4.0) and combined low pH (4.0) and Al stress, but further investigations are needed to explore the exact functions of these malonylisoflavonoids in soybean plants under various stress conditions.

### 3.4. Flavan-3-ols

**Figure 7:**
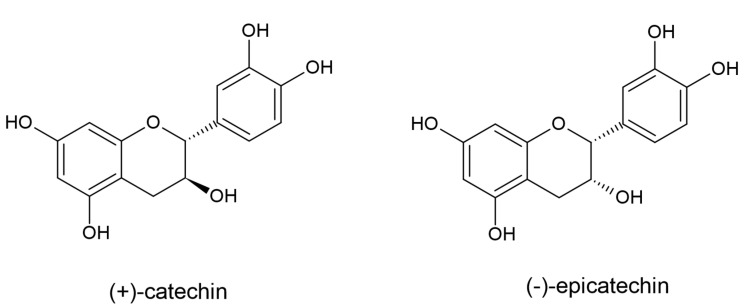
The structures of the flavan-3-ols catechin and epicatechin.

Flavan-3-ols have a hydroxyl group attached to the C3 atom of the core flavan structure (which does not contain a keto group in the C ring). They are derived from flavanones by dihydroflavonol-4-reductase (DFR), which reduces the C4 atom in pyrane ring. The most widely known compounds from this group are catechin, epicatechin, and their glycosides (Figure 7).

Flavan-3-ols are widely known as antioxidants, as they have abilities to upregulate antioxidant enzymes and to scavenge ROS [107]. As reviewed by Aron and Kennedy [108], the generally accepted biological role of flavan-3-ols in plants relates to their protection against harmful intruders such as microbes, fungi, insects, and herbivorous animals. Interestingly, in the case of an invasive species of spotted knapweed, some plant species utilized flavan-3-ols to prevent the proliferation of neighboring plant species [109].

The role of flavan-3-ols in abiotic stress is less investigated so far. Several studies that focused on flavan-3-ols levels under drought stress reported reduced levels of flavan-3-ols in juvenal plants [110,111]; while in more mature plants, flavanol levels increased under drought stress [112,113]. Similarly, salt stress increased epicatechin levels, but decreased accumulation of catechin hydrate and quercetin compounds in wheat sprouts compared to the control [114]. This might be an indication of the crucial role of 4′, 5′-dihydroxy positions in flavonoid structures when dealing with salt stress. The accumulation of flavan-3-ols was also reported under cadmium stress [115]. In addition, flavan-3-ols may be involved in low-temperature stress response. Catechin accumulation was reported in date palm [116], evergreen *Quercus suber* [117], and *Arabidopsis* [118] at low temperatures. However, shade reduced the concentration of catechins and O-glycosylated flavon-3-ols in tea buds and leaves [21]. The exception was epigallocatechin gallate, the concentration of which was not affected significantly. This compound is an ester of gallocatechin and gallic acid, so it contains a higher number of hydroxy groups than other flavanols, which could be the reason for its stability during shade treatment. Moreover, among the detected compounds, the concentration of O-glycosylated flavon-3-ols in shaded leaves decreased more significantly than the concentration of catechins, compared to the sunlight-exposed leaves. Since catechins contain more hydroxy groups than glycosylated flavonols, the importance of the hydroxylation level of flavonoids in response to shade is imposed. As reported by Williamson et al. [44], the polymerization of flavanols may affect their antioxidant activity. Dimerization and trimerization of (epi)-catechin increased antioxidant activity, but the tetramer showed decreased activity. It was speculated that the polymerization of catechins might occur in tea leaves affected by shade treatment [21]. Integrated transcriptomic and metabolomic analysis showed that exogenous abscisic acid induced flavonoid metabolism of tea plants under drought stress [119]. In tea leaves (*Camelia sinensis*) flavanols may be present as galloyl esters: epigallocatechin gallate (EGCG), epigallocatechin (EGC), epicatechin gallate (ECG), or epicatechin [120]. The galloylation of catechins and the presence of gallocatechin groups in natural extracts seem to be important chemical properties. The galloylation of flavanols increased aqueous phase antioxidant activity, but decreased lipid phase activity [44].

### 3.5. Flavonols

**Figure 8:**
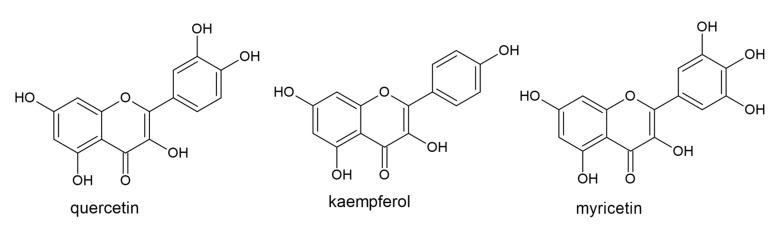
The structures of the flavonols quercetin, kaempferol, and myricetin.

Flavonols are synthesized from flavanonols by flavonol synthase (FLS), which catalyzes oxidation of C2 and C3 atoms. They have an oxidized C4 atom (a ketone group), a hydroxyl group attached to the C3 atom, and a double bond between the C2 and C3 atoms. The most commonly studied compounds in this group are quercetin, kaempferol, and myricetin (Figure 8).

Flavonols are the most emphasized flavonoid type active in stress response [121]. Historically, their role in UV-B irradiance protection is known, primarily due to their antioxidant activity. This was considered of key value during the colonization of land by plants [122]. The accumulation of different flavonols was reported under UV-B irradiance in many plant species, including Arabidopsis [123], *Capsicum annuum* [124], *Ligustrum vulgare* [125], *Vitis vinifera* [126], *Kalanchoe pinnata* [127], *Fragaria x ananasa* [128], *Mesembryanthemum crystallinum* [129], and *Ribes nigrum* [130]. Increased UV-B levels led to an increase in the ratio of quercetin and kaempferol in different cultivars of petunia [131,132], birch [133], and *Crocus* taxa [134]. The flavonols quercetin and kaempferol differ from each other only in the degree of hydroxylation on the B-ring, with quercetin being dihydroxylated and kaempferol monohydroxylated. Generally, as the level of hydroxylation increased, the absorption of UV-B decreased [133]. This is in accordance with the fact that light-responsive dihydroxy flavonoids have a much greater ability than monohydroxy to inhibit the generation of ROS, and quench ROS once they are formed [135]. In addition to UV irradiation, flavonols also mediate a plant’s response to high temperatures. For example, heat increased the level of flavonols in tomatoes (*Solanum lycopersicon* cv. Boludo) [64] and *Hypericum brasiliense* Choisy [136]. Contrary to this, in *Prunus persica*, chilling increased the flavonols [21]. This indicates the importance of flavonols in a plant’s adaptation to temperature changes.

Flavonols are relevant mediators in a plant’s response to toxic heavy metals. Cadmium increased the concentration of rutin in *Erica andevalensis* [115], while lead induced myricetin in *Prosopis farcta* 48 h after treatment, quercetin did not change, and kaempferol decreased 48 h after treatment [137]. Quercetin and kaempferol derivatives in *Vitis vinifera* were increased after treatment with titanium nanoparticles [138]. Roots of maize exposed to aluminum exuded high levels of quercetin, which suggests the ability to chelate metals as an in vivo mechanism to reduce aluminum toxicity [139]. Nanoparticles of NiO, TiO2, and Al_2_O_3_ induced quercetin and reduced kaempferol in *Nigella arvensis* [140]. Salinity is another type of stress to which plant response is mediated via flavonols, especially their glycoside forms. In tomatoes (*Solanum lycopersicon* cv. Boludo) and in *Ocimum basilicum*, it increased quercetin-3-rutinoside [17,64]. NaCl significantly increased quercetin-3-β-glucoside, but not free quercetin, in *Solanum nigrum* [135], which may indicate the involvement of glycoside form in salt-stress response. This was also confirmed in *Amaranthus tricolor* and *Solanum villosum*, in which it was observed that salt increases quercetin-3-β-glucoside [32,141].

### 3.6. Anthocyanins

**Figure 9:**
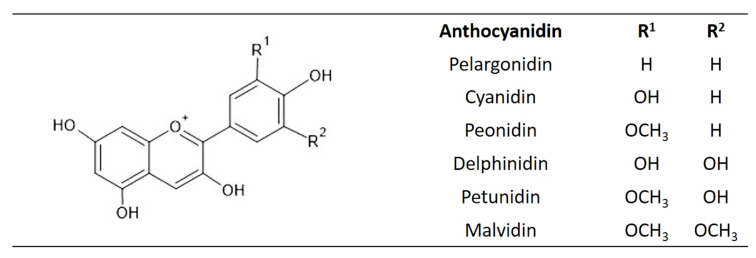
The structures of the major anthocyanidins.

Anthocyanins are group of glycosylated polyphenolic compounds of flavonoids [142,143]. They are the largest group of water-soluble pigments in the plant kingdom, and provide pink, red, orange, blue, and purple colors to different plant parts [144]. In nature, more than 600 anthocyanins have been identified [145] and classified into six categories (pelargonidin, cyanidin, delphinidin, peonidin, petunidin, and malvidin), according to the number and position of the hydroxyl and methoxyl groups on the flavan nucleus [146] (Figure 9). The main structure of anthocyanin consists of three main parts (benzyl rings A and B, C-6 and one heterocyclic benzopyran C ring, and C3) and three R groups (−H, −OH, and −OCH_3_) [147]. Glucose, galactose, rhamnose, arabinose, xylose, and glucuronic acid are the sugars frequently attached to anthocyanidins, usually as 3-glycosides and 3,5-diglycosides, or as the less common 3-diglycosides and 3-diglycoside-5-monoglycosides [148]. An important precursor to anthocyanins biosynthesis is dihydrokaempferol, which can be hydroxylated by flavonoid 3′-hydroxylase (F3′H) into dihydroquercetin, or by flavonoid 3′,5′-hydroxylase (F3′5′H) into dihydromyricetin. These two enzymes are responsible for the structural diversity of anthocyanins, and influence their B-ring hydroxylation patterns and their colors [143]. The dihydroflavonols (dihydroquercetin, dihydrokaempferol, and dihydromyricetin) are reduced by dihydroflavonol 4-reductase (DFR) to colorless leucoanthocyanidins (leucocyanidin, leucopelargonidin, and leucidelphindin). Next, the anthocyanidin synthase (ANS) catalyzes the synthesis of colored anthocyanidins from leucoanthocyanidins. Flavonoid 3-*O*-glucosyltransferase (UFGT) or other members of the glycosyltransferase enzyme family are responsible for binding sugar, and acyltransferases are responsible for binding acyl groups to anthocyanidins [143].

Anthocyanins have roles in pollination and seed dispersal, plant development, and the adaptation of plants to the biotic (pathogen attack) and abiotic (salt, drought, UV, blue light, high-intensity light, and sugar and nutrient deficiency) stress conditions [143]. Several recent studies that combined transcriptomic and metabolomic data pointed to their important role in abiotic stress responses [149,150,151]. Under stress conditions, they serve as ROS scavengers, photoprotectants, and stress signals [152,153]. The common model plant *Arabidopsis thaliana* accumulated over 20 anthocyanins derived from cyanidins [154]. Numerous studies of this plant showed the involvement of anthocyanins in stress responses to temperature (high or low), salt, and drought [154,155,156,157,158,159,160]. Stiles et al. [161] investigated the influence of temperature on eight cyanidin-based, six petunidin-based, two delphinidin-based, and one peonidin-based anthocyanins in the floral extracts of *Plantago lanceolata*. All of the isolated anthocyanins originated from the cyanidin and delphinidin branches of the anthocyanin biosynthetic pathway. Thirteen individual anthocyanins increased at cool temperatures, and four petunidin-based anthocyanins were temperature-insensitive, but the authors reported a small probability that the temperature-insensitive anthocyanins would all be from the petunidin group.

Kovinich et al. [154] showed that under various stress conditions, Arabidopsis not only accumulates significantly higher levels of total anthocyanins often, but also favors the accumulation of different sets of anthocyanins, suggesting that the various structural patterns on an anthocyanin backbone actually impart a function favorable in a particular stress condition. Unfortunately, in the study, the authors only used high-performance liquid chromatography coupled with a photodiode array detector (HPLC-PDA), which did not allow them exact structure elucidation of anthocyanins, so the exact role of the anthocyanins’ structure in abiotic stress response remains unclear.

## 4. Stilbenoids

**Figure 10:**
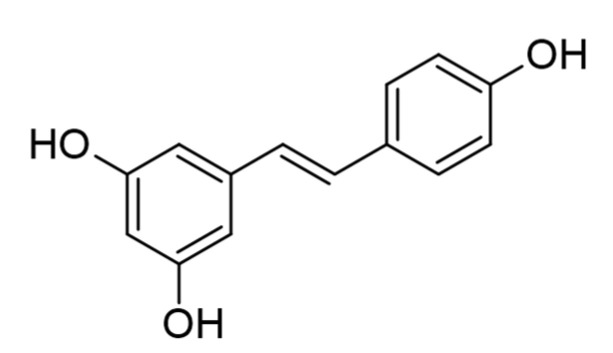
The structure of the stilbenoid resveratrol.

Stilbenoids are hydroxylated derivatives of stilbene. The essential structural skeleton of stilbene comprises two aromatic rings joined by a methylene bridge (Figure 10). Double bond in the structure do not allow free rotation, so there are only two possibilities for the stilbene configuration: *trans* or *cis* configuration. The predominant naturally occurring stilbenes have the *trans*-(E) configuration. From this relatively simple structure, nature has contrived a bewildering arrangement of hydroxyls through which these groups are substituted with sugars, methyl, methoxy, and other residues; the steric configuration of chemically identical structures; and their ability to form dimers, trimers, or larger polymers [162]. In stilbenoid synthesis in plants, the most important enzyme is stilbene synthase (STS) [163].

The role of stilbenoids in biotic stresses has been commonly investigated, and their antifungal, antibacterial, and antiviral activity are well documented [164,165,166]. The most commonly studied stilbenoid is resveratrol (Figure 10) and its derivatives, especially regarding antioxidant activity [164]. In vitro studies showed that the antioxidant activity of resveratrol, oxyresveratrol, pinosylvin, and pterostilbene was closely related to the number of their hydroxy groups, so the strongest antioxidant and free-radical scavenging activities showed oxyresveratrol, with four hydroxyl groups, and the lowest pterostilibene, with one hydroxyl group [166]. In vitro biological activity of stilbenoids could be related to their role in stress responses. Treatment of cell cultures with hydrogen peroxide (H_2_O_2_), a molecule in plants that mediates responses to stresses, and methyl jasmonate (MeJA), a key compound involved in defense-related signal transduction pathways in plants, induces the biosynthesis of resveratrol, piceatannol, and viniferins in hairy roots of *Vitis rotundifolia* [167,168]. Resveratrol accumulation was observed under abiotic stressors such as UV light, ozone exposure, or metal treatments [164,169,170]. Under stress conditions, such as downy mildew (*Plasmopara viticola*) infection, ultraviolet light, and AlCl_3_ treatment, the enzyme o-methyltransferase is activated, which catalyzes biosynthesis of pterostilbene, a methyl ether of resveratrol [171]. Resveratrol has three hydroxyl (−OH) groups, while pterostilbene has two methoxy (–OCH_3_) groups and one −OH group. Several other studies showed stronger pharmacological properties in pterostilbene than in resveratrol [172]. In addition, pterostilbene is five to 10 times more fungitoxic than resveratrol in vitro [171]. This may be attributed to its two –OCH_3_ groups, which may contribute to biological activity [172].

The defense responses of peanut (*Arachis hypogaea*) to biotic and abiotic stresses included the synthesis of prenylated stilbenoids such as arachidin-1, arachidin-2, arachidin-3, and isopentadienyl trihydroxystilbene [173]. For example, co-treatment of the hairy root culture of *Arachis hypogaea* with ethyl-β-cyclodextrin, methyl jasmonate, hydrogen peroxide (H_2_O_2_), and magnesium chloride induced prenylated stilbenoids arachidin-1, arachidin-2, arachidin-3, and arachidin-5 [174]. In general, prenylation of aromatic compounds plays an important role in the diversification of plant secondary metabolites and contributes to the enhancement of the biological activity of polyphenolic compounds [175]. Consequently, prenylated stilbenoids also have shown equivalent or enhanced bioactivities relative to non-prenylated forms, such as resveratrol, in in vitro studies [176]. The prenylation of stilbenoids increases their lipophilicities and membrane permeability, and may have additional impacts on bioactivities.

The dimeric structures of stilbenoids may also be involved in plant stress responses. Research showed that the level of resveratrol dimers, pallidol, trans-ε-viniferin, and trans-δ-viniferin increased in cell suspension of *Vitis labrusca* upon treatments with methyl-β-cyclodextrins and methyl jasmonate [177]. δ-Viniferin, a resveratrol dehydrodimer and an isomer of ε-viniferin, is one of the major stilbenes synthesized by stressed grapevine leaves [178]. This compound has been reported as a molecule produced in vitro by the oxidative dimerization of resveratrol by plant peroxidases or fungal laccases [178]. The dimeric structures of stilbenoids are reported to be involved in biotic stress [179], but their possible involvement in abiotic stress remains unknown.

## 5. Lignans

**Figure 11:**
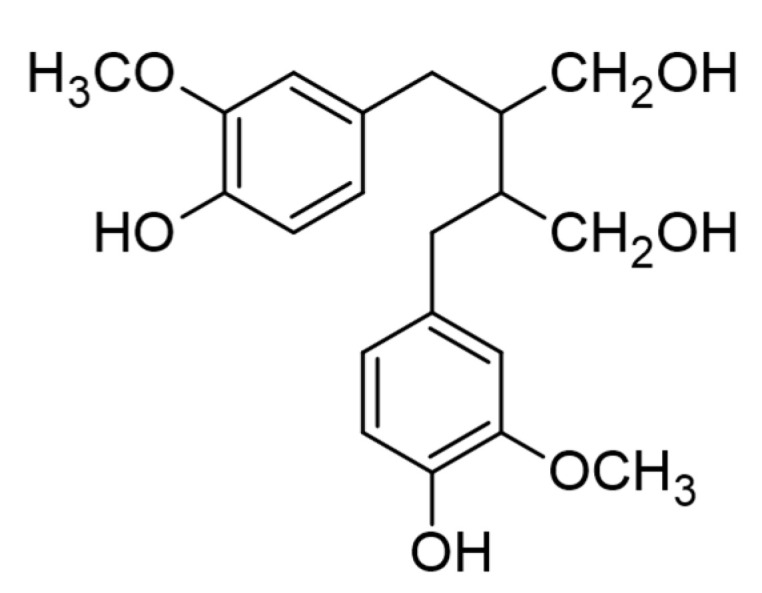
The basic lignan chemical structure.

Lignans are a large group of naturally occurring non-flavonoid, dimeric phenylpropanoids widely spread within vascular plants. Lignans contain two phenylpropanoid monomers linked by a bond between carbons C8 and C8′ [180]. Classical lignans showed dimeric structures formed by a β, β′ (or 8-8′)-linkage between two propenylphenyl units with a different degree of oxidation in the side-chain and a different substitution pattern in the aromatic moieties (Figure 11) [181]. The C3 side chains of the monomers are linked by C-C bonds tail-to-tail and head-to-tail in lignans and in neolignans, respectively. Based on structural patterns, including their carbon skeletons, the way in which oxygen is incorporated into the skeletons and the cyclization pattern, lignans are classified into eight groups: furofuran, furan, dibenzylbutane, dibenzylbutyrolactone, aryltetralin, arylnaphthalene, dibenzocyclooctadiene, and dibenzylbutyrolactol, while neolignans contain 15 subtypes known as NL1 to NL15 [181]. Most of the lignans in plants occur in a free form, while some can form glycosides and other derivatives. Numerous oxidative transformations of lignans have been reported [182], resulting in high diversity of lignan forms and compounds with different biological activities. More than 200 classical lignans and 100 neolignans have been characterized in different vascular plants.

The origin of the lignan and neolignan biosynthetic pathways is amino acid phenylalanine, a precursor of coniferyl alcohol [181]. An enantioselective dimerization of coniferyl alcohol is mediated by oxidases such as peroxidases or laccases, with the assistance of dirigent proteins (DIRs), resulting in the lignan pinoresinol, which can be further converted to other lignans (piperitol, laciresinol, sesamin, and secoisolaresinol) and their glucosides by catalytic action of specific enzymes.

Structurally close compounds to lignans in which the oxygen etheric linking is present are referred to as neolignans. There are also sesquilignans and sesquineolignans, which are structurally familiar compounds that contains three phenylpropanoid subunits linked together by carbon-carbon or carbon-oxygen bonds, as well as norlignans, which co-occur with lignans or neolignans and possess a C15, C16, or C17 core structure. Lignans could, serve as a storage pool for lignin biosynthesis. Lignin is a highly branched polymer that primarily consists of *p*-hydroxyphenyl (H), guaiacyl (G), and syringyl (S) units formed by the oxidative coupling of *p*-coumaryl, coniferyl, and sinapyl alcohols, respectively. Lignin is component of cell walls, ensuring hardness, mechanical support, and impermeability in plant tissues, and they also play an important role in defense against biotic and abiotic stress factors [183].

Similar to many other polyphenolic compounds in plants, lignans are reported to have an ecological role in helping plants to cope with biotic and abiotic stresses during growth and development [3,184]. Some of them are considered pharmacologically interesting due to their antiviral, antibacterial, antioxidant, and antitumor properties [185]. Due to their structure, lignans are effective antioxidants, and they have the potential to scavenge a harmful ROS that is usually overaccumulated under stress conditions. In nature, oxidations, mostly related to biosynthetic pathways, are processes in which lignans act as a primary antioxidant by scavenging free radicals. The antioxidant activity of many lignans was evaluated and confirmed in a variety of in vitro assays. The flaxseed lignan secoisolariciresinol diglycoside (SDG), and its mammalian metabolites enterodiol (ED) and enterolactone (EL), were reported to possess antioxidant but no prooxidant activity [186,187]. SDG, ED, and EL were similarly effective at lowering lipid peroxidation, while ED and EL displayed greater efficacy in reducing deoxyribose oxidation and DNA strand breakage. Suja et al. [188] screened the antioxidant activity of isolated and purified compounds from sesame cake: sesamol, sesamin, sesamolin, sesaminol diglucoside, and sesaminol triglucoside. The results of the β-carotene-bleaching assay and the inhibition of linoleic acid peroxidation by the thiocyanate method showed that the examined lignans possessed antioxidative activity to different extents, depending on their structure. The order of investigated lignans based on antioxidant activity was as follows: sesamol > sesamolin ≥ sesamin > sesaminol triglucoside > sesaminol diglucoside. Glucosides showed less antioxidant power compared to free-form lignans. Lignans isolated from root bark of fringe tree (*Chionanthus virginicus* L.), such as phillyrin, pinoresinol-β-d-glucoside (PDG), and pinoresinol di-β-d-glucoside (PDDG), showed significant antioxidant activity compared to standard antioxidants (butylated hydroxyanisole (BHA), butylated hydroxy-toluene (BHT), and α-tocopherol and its water-soluble analogue trolox) in a series of in vitro tests [189]. Based on in vitro assays, it was found that an oxygen-free benzylic position is important in the higher radical-scavenging activity of butane-, trahydrofuran-, and butyrolactone-type lignans [190].

The antioxidant activity of lignans is a basic feature of their positive role in plant abiotic stress responses and tolerance. Furthermore, their involvement in lignification and cell-wall synthesis also has a potentially positive role in stress responses. Drought stress may change the level of lignans in the main sesame (*Sesamum indicum* L.), depending on genotypes. In fact, more drought-tolerant genotypes exhibited higher levels of sesamin and/or sesamolin [191]. It is interesting that light-colored sesame seeds contained higher sesamin and sesamolin than dark-colored seeds. It was shown that upregulation of lariciresinol biosynthesis in *Isatis indigotica*, particularly in tetraploids compared to diploids, improved root development and enhanced salt and drought stress tolerance [192]. A key enzyme of lignan biosynthesis in *Isatis indigotica*, 4-coumarate:coenzyme A ligase 3 (Ii4CL3), was shown to be activated by transcription factor IiWRKY34. IiWRKY34 expression, which is significantly higher in tetraploids than in diploids, is positively correlated with lariciresinol accumulation and the better performance of plants under stress conditions.

Experiments on metal stress showed that CB671 (tolerant Cd-accumulating) genotype of oilseed rape (*Brassica napus*) accumulates lignans along with cell-wall saccharides, evoking cell-wall priming compared to the sensitive Cd-accumulating genotype (ZD622) that markedly accumulates phenolics from upstream subclasses of flavonoids [193]. The observed accumulation of lignans, along with the accumulation of cell-wall saccharides in Cd-tolerant *B. napus* genotype, could imply an enhanced antioxidant capacity in the cell wall and an active involvement of the cell wall in countering the stress imposed by an elevated Cd level.

As previously mentioned, DIRs are important proteins in lignans, and in mediating the regio- and stereo-selectivity of bimolecular coupling during lignan biosynthesis. DIRs and peroxidases have been reported to be involved in the modulation of lignification levels upon exposure to abiotic stress [184]. For example, Mn toxicity caused up-regulation of PEROXIDASE5- and DIR2-like proteins and enhanced the cell-wall lignification in soybean roots; some DIR genes were up-regulated by heat stress in *M. sativa*; and enhanced expression levels of TaDIR were reported under salinity–alkalinity stress in *Tamarix androssowii*. In pepper (*Capsicum annuum* L.), the silencing of CaDIR7 caused a significant decrease in the chlorophyll content in leaf discs exposed to NaCl and mannitol (300 mM each) (56.25% and 48%, respectively) [194], suggesting a protective role of CaDIR7 protein in abiotic stress.

In addition to their role in abiotic stress response, lignans play an important part in plants’ defense against pathogens by inhibiting microbe-derived degradative enzymes such as cellulases, polygalacturonases, glucosidases, and laccases. Furthermore, lignans can function as insecticides due to their disruption of the insect endocrine system [184].

Biotic and abiotic stress conditions are usually connected with increased levels of stress hormones (salicylic acid, abscisic acid, jasmonic acid). It was shown that exogenous treatments with stress hormones might enhance lignan biosynthesis. Thus, an addition of salicylic acid (50 μM) in cell culture of flax (*Linum usitatsimum* L.), resulted in a two- to four-fold higher level of biosynthesis of lignans (secoisolariciresinol diglucoside (SDG) and lariciresinol diglucoside (LDG)) and neolignans (dehydrodiconiferyl alcohol glucoside (DCG) and guaiacylglycerol-β-coniferyl alcohol ether glucoside (GGCG)) compared to the control [195]. Furthermore, the induction of lignan biosynthesis by methyl jasmonate, both at the gene-expression and metabolite-accumulation levels (coniferin, lariciresinol, secoisolariciresinol, and pinoresinol) was demonstrated in *Isatis indigotica* hairy root cultures [196]. Elicitation of phenypropanoid biosynthesis with stress hormones is becoming a popular biotechnological strategy in the production of pharmacologically valuable polyphenols, including biological active lignans.

## 6. Conclusions and Further Research

Polyphenols play a crucial role in plant–environmental interactions. In our review, we summarized data from the available literature on how phenolic acids, flavonoids, stilbenoids, and lignans are involved in abiotic stress responses. Their bioactivity and their role in stress defense are commonly attributed to their antioxidant activity, mainly supported by the in vitro data. For example, antioxidant activity of phenolic acids is mainly related to the number of hydroxyl groups, and HCAs are often more effective antioxidants than HBAs. However, it is difficult to offer unambiguous conclusions, because antioxidant activity in vitro also depends on experimental conditions, used assays, etc. Other biological roles of polyphenols in stress responses, tested in vivo, also depend on experimental conditions such as the duration of exposure to stress, the chosen model plants, the plant growth stage, and the method of analyzing metabolites. Numerous studies of in vivo plant stress responses reported changes only in the total amount of polyphenols or the changes in specific group, without knowing the exact molecules, which change under the stress. Consequently, according to available literature, it is difficult to conclude exactly which polyphenol structures are involved in specific protective mechanisms, despite some studies that showed that this may be the case. The reason could be that these types of studies require a multidisciplinary approach and the use of modern metabolomics platforms, especially the integration of metabolomics and transcriptomic data. Recently, these methods have become more accessible, and it is expected that future studies will better explain how polyphenol structures influence their role in plant stress responses.

## Data Availability

No new data were created or analyzed in this study. Data sharing is not applicable to this article.
